# Evaluation of the Population Genetic Structure of *Anadara tuberculosa* (Mollusca, Bivalvia) in the Panamanian Pacific

**DOI:** 10.1002/ece3.73396

**Published:** 2026-04-22

**Authors:** Thalia Garcia, Carmen Mela‐Sánchez, Celestino Aguilar, Zeuz Capitan‐Barrios, Angel Vega, Leyda Abrego, Yolani A. Robles P.

**Affiliations:** ^1^ Centro de Capacitación, Investigación y Monitoreo de la Biodiversidad en el Parque Nacional Coiba (CCIMBIO‐CRUV‐UP) Universidad de Panamá Santiago Veraguas Panamá; ^2^ Facultad de Ciencias y Tecnología Universidad Tecnológica de Panamá Panama City Panamá; ^3^ Facultad de Medicina Universidad de Panamá Panama City Panama; ^4^ Facultad de Ciencias Naturales, Exactas y Tecnología Universidad de Panamá Panama City Panama

**Keywords:** cytochrome oxidase I, genetic connectivity, genetic structure, haplotype network, ocean currents

## Abstract

*Anadara tuberculosa* is a bivalve mollusk common in the mangroves of the Panamanian Pacific, subject to commercial exploitation throughout its distribution area. Fishing pressure, combined with environmental contamination processes, is a factor that can influence the genetic structure of the species. Understanding the genetic aspects of *A. tuberculosa* is relevant for developing conservation policies; therefore, this study analyzed the genetic diversity, structure, and demographic history of this species in five localities of the Panamanian Pacific using the mitochondrial COI gene. The results revealed high haplotypic (0.93–0.97) and low nucleotide (0.0076–0.0095) diversity with generally low genetic differentiation, although significant structure was detected specifically between Coiba and Isla Cañas. Demographic analyses and a star‐shaped haplotype network indicate a recent population expansion, a signal that was most pronounced in the Chame locality. The low genetic differentiation is attributed to the presence of coastal currents and geographical barriers, which together shape each site and influence larval dispersal and cause some degree of genetic connectivity. This study provides a preliminary genetic baseline that can support the design of site‐specific management actions and periodic genetic monitoring aimed at preserving diversity and contributing to the long‐term sustainability of this essential resource for harvesting communities and the ecological processes occurring in the mangrove ecosystems of the studied localities.

## Graphical abstract

Our study analyzed the population genetics of *Anadara tuberculosa* (concha negra) in five Panamanian Pacific mangrove areas using the mitochondrial COI gene. We found high haplotypic diversity and low nucleotide diversity, a pattern that suggests a recent demographic expansion, which was supported by our neutrality tests and a star‐shaped haplotype network. The results indicate a generally high level of genetic connectivity among populations, with the exception of the Coiba and Isla Cañas populations, which showed significant genetic differentiation likely due to geographic barriers and ocean currents.

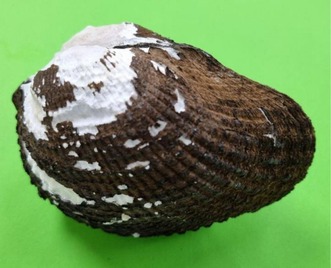

## 1 Introduction

**FIGURE 1:**
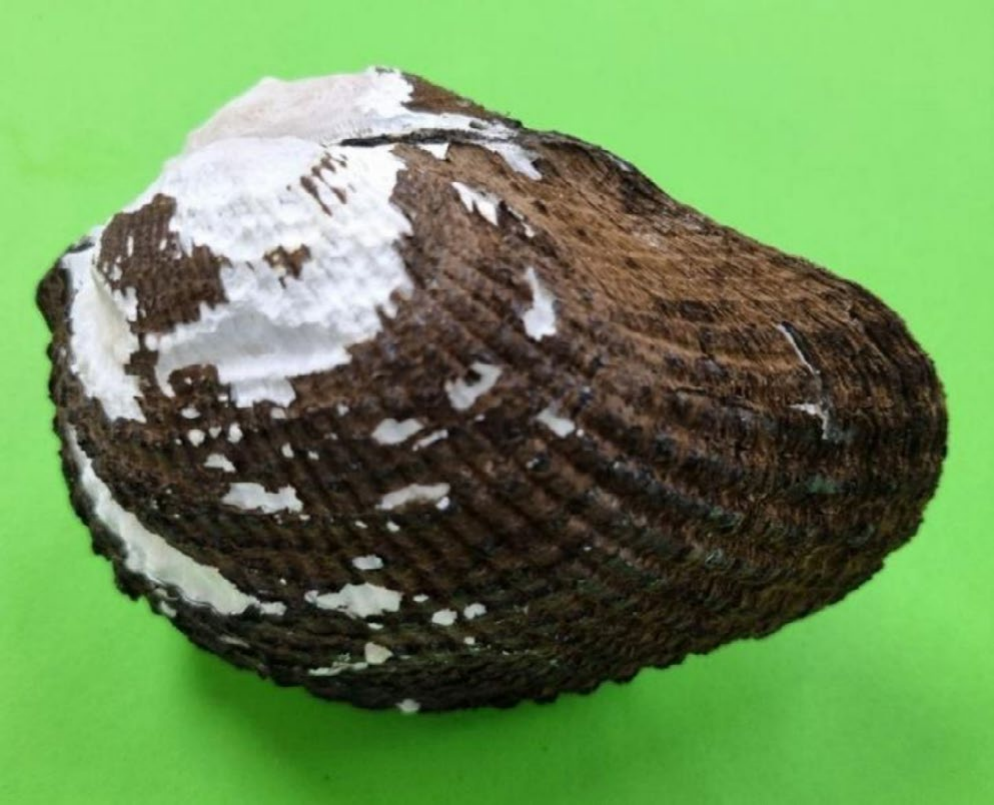
*Anadara tuberculosa* (Concha negra) is a species of fishery importance in the Eastern Pacific. It is characterized by a thick, coarse shell with prominent radial ribs and inhabits the organic‐rich, muddy substrates of tropical mangrove ecosystems, where it lives in a buried state.

*Anadara tuberculosa* (Sowerby, 1833) (Figure 1) is a bivalve mollusk of the Arcidae family (Keen 1971), known as “concha negra” (mangrove cockle) in Panama, has a planktonic larval stage of about 21–30 days (Borda and Cruz 2004; Diringer et al. 2019), and inhabits mangrove ecosystems where it plays a fundamental ecological role through water filtration, contributing to the removal of suspended particles (Wong et al. 1997). Its ability to adapt to variable environmental conditions such as temperature, salinity, and food availability allows it to persist in this complex and dynamic environment (Baqueiro Cárdenas and Aldana Aranda 2003; Vega et al. 2021). It is a resource exploited throughout its distribution area (MacKenzie Jr 2001), with significant indicators of overexploitation in countries such as Costa Rica (Stern‐Pirlot and Wolff 2006), Colombia (Lucero et al. 2012), Ecuador (Prado‐Carpio et al. 2021), and Peru (Ordinola et al. 2019). The increase in capture linked to the decrease in mangrove areas has led to a reduction in population density (Mora and Moreno 2009; Vega et al. 2021), which promotes the loss of genetic diversity (Charlesworth 2003), decreasing the adaptive response and survival of a population (Reed and Frankham 2003).

Studies on the genetic diversity of the mangrove cockle *Anadara tuberculosa* are scarce, and the few that exist report a reduction in diversity, evidenced by a loss of heterozygosity (Fuentes et al. 2024). This decline has been tentatively linked to increased inbreeding associated with recent overexploitation events (Fuentes et al. 2024).

Furthermore, the existence of different lineages within *A. tuberculosa* has been reported, suggesting local adaptations to specific environmental conditions (Chamorro and Rosero 2016). A comparative study across Colombia, Ecuador, and Peru demonstrated a high level of genetic variation between populations situated north and south of the Equator, which was attributed to the species' life cycle and ocean current patterns (Diringer et al. 2019).

In Panama, *A. tuberculosa* is distributed along the Pacific coast (MacKenzie Jr 2001; Gomez et al. 2023), with vital extraction and commercialization sites located in the mangroves of David and the Gulf of Montijo. Within these Panamanian populations, a decrease in population densities, variations in sex ratio, and the appearance of hermaphroditism have been documented, likely associated with exploitation and/or contamination factors (Vega et al. 2021; Robles‐P. et al. 2022).

Specifically, Robles‐P. et al. (2022) reported these reproductive alterations in Panamanian populations, which may be related to environmental stress. They considered the presence of hermaphroditism a non‐casual, intrinsic, and recent process in the species, possibly emerging as a population response to declining densities (Robles‐P. et al. 2022). The incidence of hermaphroditism has critical implications for genetic diversity, as reproductive alterations—particularly self‐fertilization events—can significantly reduce genetic diversity. For instance, in the hermaphroditic bivalve *Argopecten irradians irradians* , self‐fertilization events can reduce population genetic diversity by between 10% and 40% in a single generation (Zheng et al. 2008, as summarized by Barros et al. 2020). Therefore, the reported incidence of hermaphroditism in Panamanian *A. tuberculosa* populations could contribute to a long‐term loss of genetic diversity in the species.

The population changes in *A. tuberculosa* underscore the need to understand the genetic diversity, structure, and evolutionary history of this species for its conservation. This facilitates devising management strategies based on scientific evidence that ensure the stability of populations and their ecological functions (Zamora‐Bustillos et al. 2011; Diringer et al. 2019). In recent decades, the rise of nucleic acid sequencing technologies has allowed biological research to be redirected towards a deeper understanding of genetic variability and the evolutionary processes that shape natural populations. The use of molecular markers has become a widely used tool in genetic studies to understand genetic diversity, genetic structure, demographic history, and connectivity patterns, as well as to support the design of conservation strategies for ecologically and economically important species (Galtier et al. 2009). Based on this, we proposed to analyze the diversity and genetic structure, as well as the phylogenetic relationships of *A. tuberculosa* in five localities of the Panamanian Pacific, using a segment of the mitochondrial gene of Cytochrome C Oxidase subunit I (COI) as a molecular marker.

Considering coastal geography and regional current patterns, a certain degree of connectivity among mainland populations might be expected. However, the geographic distance between sites and the discontinuous distribution of mangrove habitats may favor genetic structuring. Therefore, rather than assuming a priori uniformly high gene flow, we hypothesized the existence of intermediate connectivity among mainland populations, contrasted by distinct genetic differentiation in the insular population of Coiba.

## 2 Materials and Methods

### 2.1 Sampling and Processing

**FIGURE 2:**
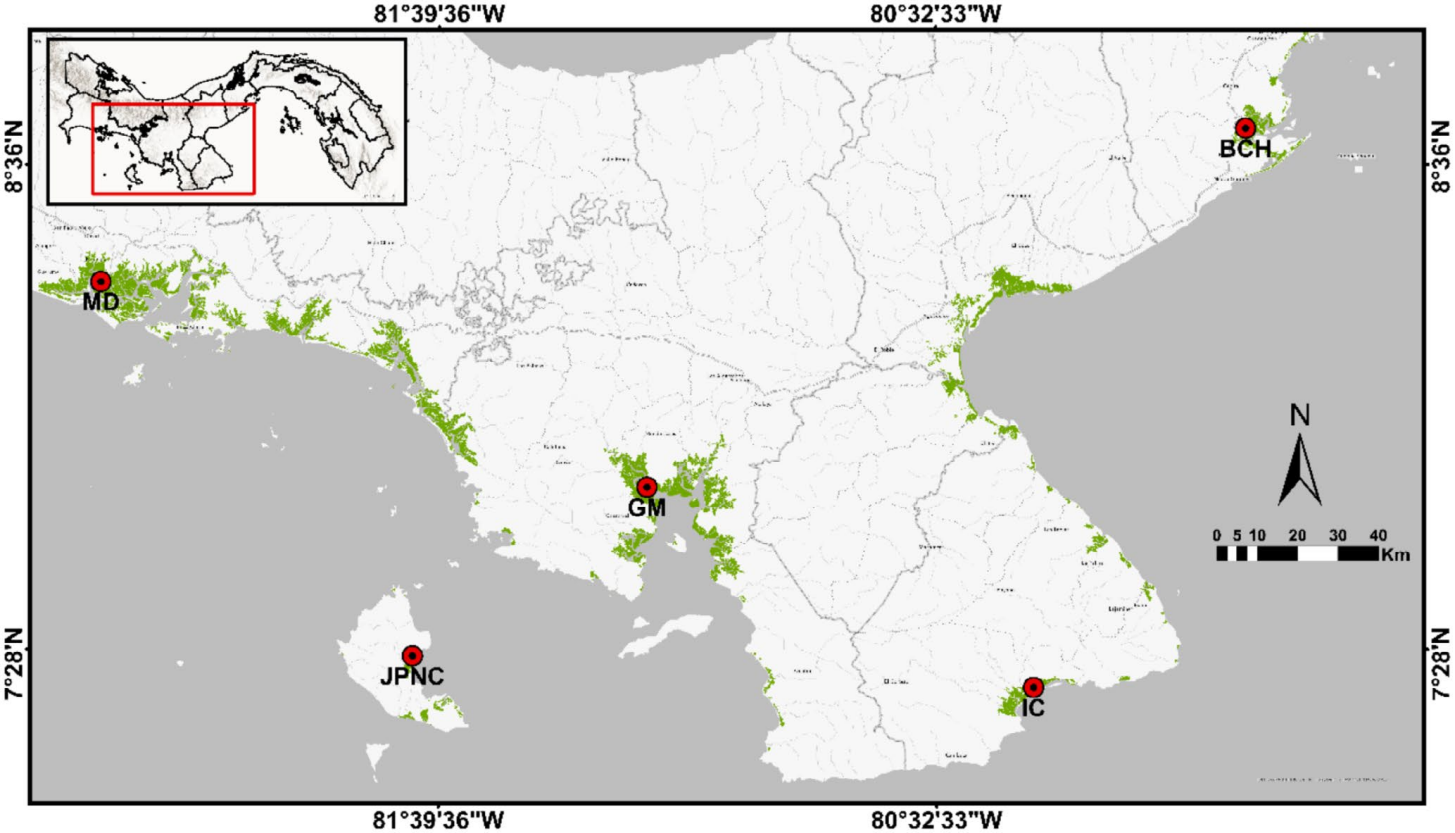
Collection sites for *Anadara tuberculosa* samples in the Panamanian Pacific. MD: Mangroves of Chame Bay (8°39′49″ N, 79°50′52″ W, and 8°39′35″ N, 79°50′48″ W), Isla Cañas (7°22′45″ N, 80°15′30″ W, and 7°25′45″ N, 80°21′32″ W), Juncal in Coiba National Park (7°29′07.97″ N, 81°43′08″ W, and 7°29′14″ N, 81°43′11″ W), Gulf of Montijo (7° 10′ 4″ N, 81° 32′ 35″ W, and 7° 53′ 27″ N, 81° 56′15″ W), and Mangroves of David (8°16′59.98″ N, 82°30′25.88″ W, and 8°14′27.00″ N, 82°18′15.67″ W). Source: Spatial analysis unit of the CCIMBIO‐CRUV‐UP.

Specimens of mangrove cockle were collected manually in five mangrove areas of the Panamanian Pacific during 2023 and 2024. The collection sites were: Mangroves of Chame Bay, Isla Cañas, Juncal in Coiba National Park, Gulf of Montijo, and Mangroves of David (Figure 2). The specimens were transported to the wet laboratory of the Center for Biodiversity Training, Research, and Monitoring in Coiba National Park, located at the Universidad de Panamá, Veraguas campus, for processing. The mangrove cockle is commercially extracted in all these mangroves, except in Coiba National Park (Vega et al. 2021; Robles‐P. et al. 2022; Del Cid‐Alvarado et al. 2024).

### 2.2 DNA Extraction, Amplification, and Sequencing

In the laboratory, the specimens were washed, dried, and opened to extract tissue from the foot, adductor muscle, or mantle, which was preserved in absolute ethanol and stored at −20°C. DNA extraction was performed using the E.Z.N.A. Mollusk DNA kit (Omega Bio‐Tek). For amplification, primers LCO1490: 5′‐ggtcaacaaatcataaagatattgg‐3′ and HC02198: 5′‐taaacttcagggtgaccaaaaaatca‐3′ from Folmer et al. (1994) were used following the protocol of Diringer et al. (2019) with slight modifications, such as an initial denaturation for 5 min at 95°C, then 45 cycles of denaturation for 30 s at 94°C, annealing for 45 s at 50°C, followed by extension for 1 min at 72°C, and a final extension step of 7 min at 72°C, to obtain a 620 bp sequence of the COI gene. Electrophoresis on a 1.5% agarose gel allowed visualization of the amplified fragments with a MyGel InstaView Mini. Sequencing was performed using the Sanger method on a 3130xl Genetic Analyzer after purification with the XTerminator method.

### 2.3 Genetic Diversity and Genetic Structure

The sequence chromatograms were edited with Sequencher 4.1.4 software (Nishimura 2000) and verified in GenBank using BlastN. The generated consensus sequences were aligned with MAFFT (Katoh et al. 2019) and edited using Geneious Prime v2025.1.2 (Kearse et al. 2012). The number of haplotypes and polymorphic sites was determined using DnaSP v6 (Rozas et al. 2017) to estimate the number of haplotypes (N), which indicates the quantity of unique genetic variants; haplotype diversity (h), which measures the probability of finding distinct haplotypes between two random individuals; and nucleotide diversity (π), which reflects the average per‐site difference between sequences. To evaluate the population structure, an Analysis of Molecular Variance (AMOVA) was performed using Arlequin v3.5.18 (Excoffier and Lischer 2010), and pairwise F_ST_ values were calculated to estimate genetic differentiation between sampling locations. Additionally, Tajima's *D* and Fu's Fs neutrality tests were applied to detect signs of population expansion or contraction. Finally, a haplotype network was constructed with PopART v. 1.7.2 (Leigh et al. 2015), using the TCS algorithm (Clement et al. 2000). The haplotype networks were color‐coded by region to visualize the geographic distribution of haplotypes.

### 2.4 Phylogenetic Analysis

The 180 *Anadara tuberculosa* sequences obtained in this study were aligned using MAFFT (Katoh et al. 2019). The Maximum Likelihood (ML) phylogenetic tree of the partial COI region was generated using IQ‐TREE version 2.2.0 (Minh et al. 2020), first determining the best substitution model using ModelFinder (Kalyaanamoorthy et al. 2017) and calculating 1000 ultra‐fast bootstraps to assess branch support. Finally, the phylogenetic tree was viewed and edited using FigTree v1.4.4.

## 3 Results

### 3.1 Genetic Diversity and Population Structure

The analysis of 180 sequences revealed the presence of 42 unique haplotypes distributed among the five sampled localities of the Panamanian Pacific, with multiple haplotypes per locality and a considerable proportion of exclusive haplotypes. Haplotype diversity (Hd) values were consistently high in all populations, ranging from 0.935 (Coiba) to 0.971 (Chame). Nucleotide diversity (π) was relatively low at all sites, with values between 0.0076 (Montijo) and 0.0095 (Isla Cañas). Chame stood out with the highest number of haplotypes (*h* = 24) and the highest haplotype diversity (Hd = 0.97143). Genetic diversity indices for all sampled populations are presented in Table 1.

**TABLE 1:** Genetic diversity indices and neutrality test statistics for *Anadara tuberculosa* in five Panamanian Pacific mangroves.

Population	*n*	*h*	Hd	π	Np	*S*	Tajima's *D*	Fu's Fs
Coiba	36	19	0.935	0.0077	8	27	−1.245	0.473
Montijo	36	19	0.954	0.0076	7	25	−1.132	0.232
Chame	36	24	0.971	0.0095	3	40	−1.668^a^	−4.3
David	36	17	0.962	0.0089	0	26	−0.864	0.808
Isla Cañas	36	19	0.96	0.0095	0	30	−1.033	−0.769

*Note:* Neutrality test statistics (Tajima's *D* and Fu's Fs) are shown.

Abbreviations: π = nucleotide diversity, *h* = number of haplotypes, Hd = haplotype diversity, *n* = sample size, S = number of segregating (polymorphic) sites.

^a^ Statistically significant *p*‐value (*p* < 0.05).

The Analysis of Molecular Variance (AMOVA) revealed a weak overall genetic structure among the localities, with a global *F*_ST_ value of 0.0082. Most pairwise comparisons were not significant; however, a statistically significant genetic differentiation was found between the Coiba and Isla Cañas populations (*F*_ST_ = 0.035, *p* < 0.05), see Table 2.

**TABLE 2:** Pairwise *F*_ST_ values among five populations of *A. tuberculosa* .

Population	Coiba	Montijo	Chame	David	Isla Cañas
Coiba	0.000				
Montijo	−0.013	0.000			
Chame	0.034	0.026	0.000		
David	0.002	0.0005	−0.009	0.000	
Isla Cañas	0.035*	0.025	−0.015	−0.011	0.000

*Note:* Pairwise *F*_ST_ values are presented below the diagonal. Significant values (*p* < 0.05) are marked with an asterisk (*).

### 3.2 Demographic History

The results of the neutrality tests are summarized in Table 1. Demographic history was evaluated using Tajima's *D* and Fu's Fs indices. Tajima's *D* values were negative in all analyzed localities; however, only Chame showed statistically significant values for the Tajima's *D* index (−1.668, *p* = 0.029). These negative and significant results indicate that the population may have experienced a recent expansion, leading to an accumulation of low‐frequency variants. Complementarily, Fu's Fs index values were also negative in all localities; however, for this index, no locality presented statistically significant values. In particular, Chame was the locality with the lowest Fs value and a *p*‐value close to significance (Fs = −4.30, *p* = 0.086), which supports the signal detected by the Tajima's *D* index, see Table 1.

**FIGURE 3:**
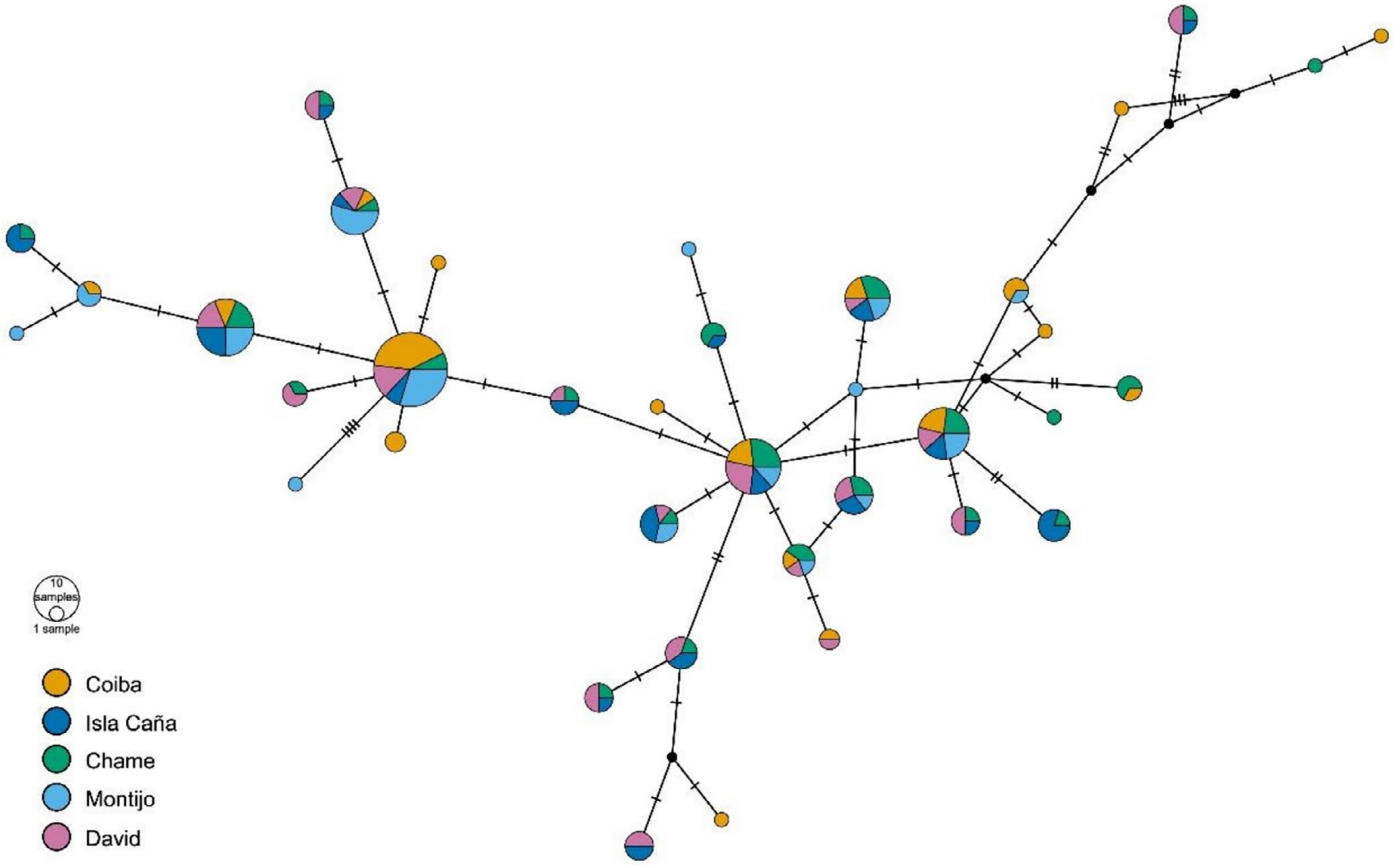
Haplotype network developed with the TCS method in PopArt. The size of each circle is proportional to the haplotype frequency. The color shows the geographical region. The bars along the lines indicate differences in the nucleotide sequences of the specimens. The black circles indicate missing intermediate haplotypes.

**FIGURE 4:**
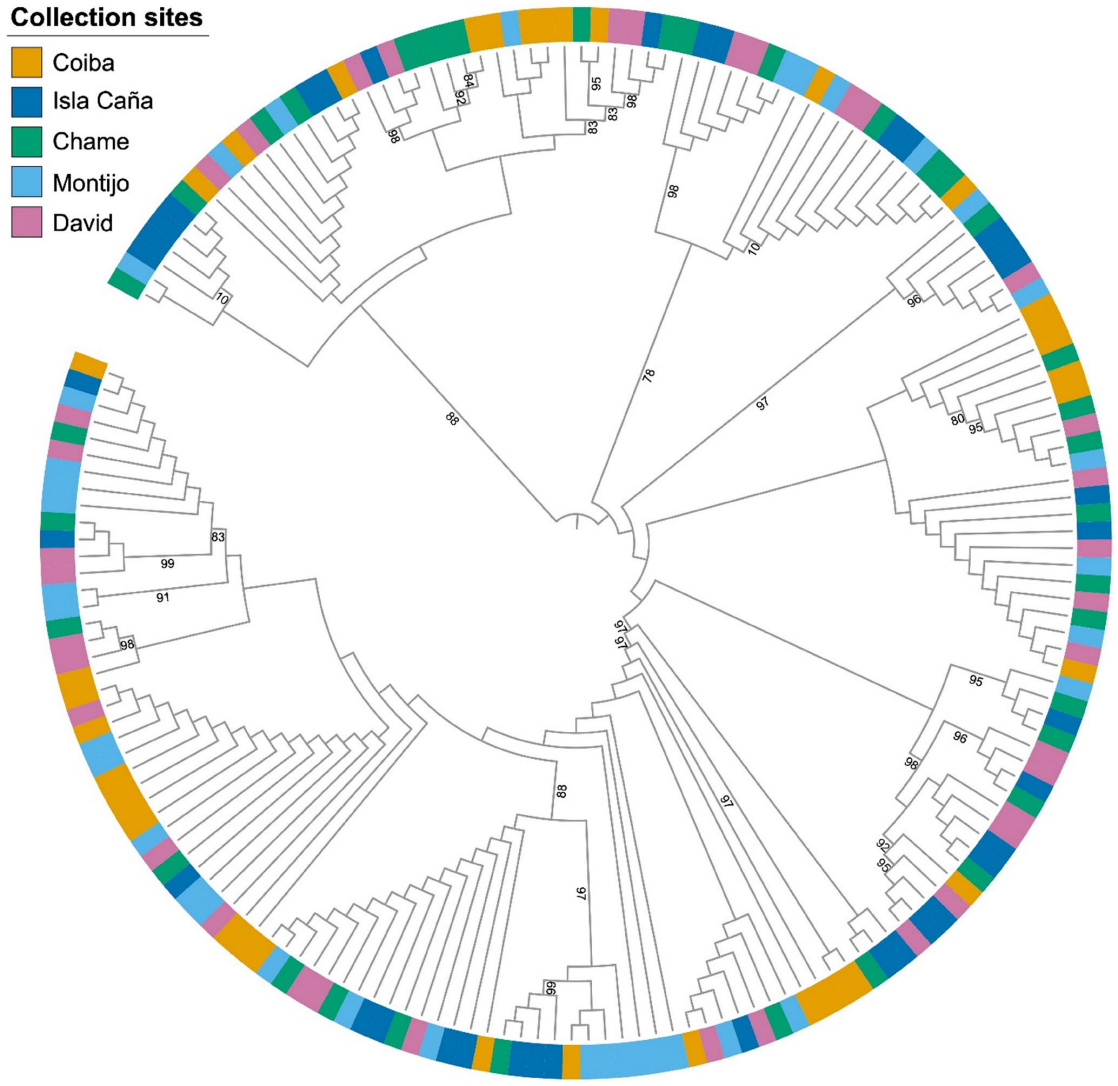
Midpoint‐rooted maximum‐likelihood phylogenetic tree constructed based on the partial COI gene sequences of 180 Panamanian *A. tuberculosa* specimens. Bootstrap values less than 70% are not shown. Green sqaure indicate Chame specimens, orange square indicate Coiba specimens, pink square indicate David specimens, blue square indicates Isla Cañas and light blue square indicate Montijo specimens.

The haplotype network (Figure 3.) showed a partially star‐like configuration, dominated by central haplotypes shared among multiple localities, and the presence of peripheral, exclusive haplotypes. The dispersion of derived haplotypes is wide, but many are only a single mutation away from the central haplotypes, which is a typical sign of expansion. The presence of unrepresented median nodes (black nodes) may be due to unsampled or extinct lineages and is consistent with processes of moderate expansion from common haplotypes. However, this lack of phylogeographic signal was confirmed by the phylogenetic analysis. The Maximum Likelihood (ML) tree derived from individual sequences displayed a scattered pattern (Figure 4), which made it difficult to resolve distinct geographic populations. The subsequent absence of significant clustering in the phylogenetic trees suggests that the *A. tuberculosa* populations lack a strong phylogeographic structure or clear geographical differentiation, indicating frequent gene flow and mixing of genetic lineages across regions.

## 4 Discussion

### 4.1 Genetic Diversity

The analysis of the mitochondrial COI gene sequence from 180 specimens of *A. tuberculosa* collected in five mangroves of the Panamanian Pacific revealed high haplotype diversity but low nucleotide diversity. High haplotype diversity combined with low nucleotide diversity is a well‐documented pattern in marine bivalves, particularly when using the mitochondrial COI gene. This marker often reveals numerous recent haplotypes separated by only a few nucleotide substitutions, resulting in shallow, star‐like genealogies (Grant and Bowen 1998; Xue et al. 2014; Hebert et al. 2016; Papadopoulos et al. 2024). Such patterns are commonly associated with species characterized by large population sizes and high larval connectivity, which promote the retention of new haplotypes while limiting deep lineage divergence. In this context, the observed Hd—π combination is widely recognized in mollusks as a signature of rapid demographic expansion following a bottleneck (Vikhrev et al. 2022; Li et al. 2025). This pattern is consistent with a history of recent demographic expansion combined with extensive gene flow, as reported for several marine bivalves including *Donax vittatus* , *Perna viridis*, *Pharella acutidens*, and *Pinctada fucata* (Fernández‐Pérez et al. 2017; Lau et al. 2018; Nasution et al. 2022; Shan et al. 2023).

The high values of haplotypic and low nucleotide diversity indicate that the populations maintain considerable genetic variability, consistent with observations in species with wide distribution, prolonged larval phases (estimated at 21–30 days in *A. tuberculosa* ), and the ability to adapt to variable conditions (Wong et al. 1997; Baqueiro Cárdenas and Aldana Aranda 2003; Lucero‐Rincón et al. 2013). This is also consistent with the findings of Chamorro and Rosero (2016) and Diringer et al. (2019), who reported high genetic diversity in *A. tuberculosa* in Tumaco (*h* = 0.859–0.962, π = 0.04) and in Colombia, Ecuador, and Peru (*h* = 0.874–0.986, π = 0.005–0.008), respectively. Furthermore, the genetic diversity values in this study equal or exceed those reported for other bivalves such as *Mytilus galloprovincialis* (*h* = 0.857, π = 0.008) and *Mytilus chilensis* (*h* = 0.943, π = 0.004) on the Chilean coasts (Oyarzún et al. 2024); and *Crassostrea gigas* (*h* = 0.423, π = 0.002) and *Crassostrea sikamea* (*h* = 0.730, π = 0.004) on the Japanese, Korean, and Chinese coasts (Sekino et al. 2012).

Chamorro and Rosero (2016) attributed the observed genetic diversity to mutation and natural selection forces, derived from the adaptive responses of individuals to natural and anthropogenic environmental changes. Therefore, it is possible that certain populations, being more exposed to environmental fluctuations and fishing exploitation, develop greater genetic variability as an adaptive response. Interestingly, this could explain why Coiba, a protected area with park status where the extraction of this resource is not permitted (Maté et al. 2009), showed the lowest genetic diversity (Hd: 0.935, π: 0.0077). The 85‐year operation of the penal colony likely caused a bottleneck due to sustained subsistence harvesting, explaining why this site exhibits the lowest diversity despite current protection. This low diversity persists because genetic recovery lags significantly behind demographic recovery. On the other hand, Sekino et al. (2012) suggest that evolutionary forces such as recurrent gene flow, habitat discontinuity, and population instability can generate patterns of high haplotypic diversity, as they observed in *Crassostrea gigas* . Finally, it is relevant to consider the potential impact of reproductive alterations reported for these populations. Robles‐P. et al. (2022) documented changes in sex ratios and the appearance of functional hermaphroditism in Panamanian *A. tuberculosa* , attributed to environmental stress. In theory, self‐fertilization associated with hermaphroditism can significantly reduce genetic diversity by decreasing the effective population size (Zheng et al. 2008). However, our study reveals consistently high haplotype diversity. This suggests that the emergence of hermaphroditism is likely a recent phenomenon—as proposed by Robles‐P. et al. (2022)—and there has not yet been sufficient evolutionary time for these reproductive changes to erode the historical genetic variability of the species. Nevertheless, if these conditions persist, a loss of heterozygosity could be expected in future generations. Together, these results suggest that *A. tuberculosa* populations in the Panamanian Pacific maintain robust genetic variability, which may favor their resilience to environmental changes and anthropogenic pressures, representing an advantage for their conservation and sustainable management.

### 4.2 Population Structure and Demographic History

In this study, the AMOVA analysis indicated that the variability within populations is greater than the variability between populations, which is consistent with the report by Chamorro and Rosero (2016), who concluded that the genetic differences were not explained by geographical distances. Regarding the genetic differentiation coefficients (*F*_ST_) that determine the genetic structure between populations, these were low on average (*F*_ST_ = 0.0082), indicating considerable gene flow among the localities. However, there were no significant differences in the genetic structure of the studied localities, except between Isla Cañas and Coiba, which suggests that local genetic isolation may exist between these sites.

Various studies point to multiple factors influencing the genetic structure of bivalve populations. Firstly, there is the capacity for gene flow, characterized by wide larval dispersal, which is accompanied by settlement preferences and larval retention mechanisms in specific microhabitats that limit gene flow (Li et al. 2025). Secondly, the presence of ocean currents, habitat fragmentation, and environmental variability also limit gene flow and lead to the establishment of local genetic structures (Li et al. 2018). Another important factor is geographical isolation, characteristic of regions with geographical barriers or environmental gradients, such as variations in salinity or temperature, which can act as ecological barriers by reducing larval survival and settlement, thereby generating greater genetic differentiation. In contrast, in coastal regions where there is no geographical isolation, genetic differentiation between populations is usually very low (Sekino et al. 2012; Li et al. 2018). Added to these are anthropogenic factors that can aggravate genetic isolation, such as overfishing, habitat modification, and pollution—pressures that alter the reproductive balance of populations, reduce effective size, and contribute to the loss of rare alleles, thereby increasing genetic vulnerability (Benavides and Carrión 2001).

Recent evidence indicates that the Colombia Coastal Current maintains a year‐round northward flow along the Panamanian Pacific, entering the Gulf of Panama (Torres et al. 2023). Given that the maximum larval dispersal potential can range between 1000 and 2000 km, depending on current velocities and settlement timing (Diringer et al. 2019), it is plausible to infer a predominant east‐to‐west gene flow connecting *A. tuberculosa* populations from Chame to David. However, distinct geographical characteristics likely influence this connectivity. Specifically, the Azuero Peninsula separates two oceanographically distinct regions—the Gulf of Chiriquí and the Gulf of Panama—characterized by spatially heterogeneous current and upwelling regimes (D'Croz and O'Dea 2007). Previous studies suggest that such peninsular geomorphology, combined with wind and tidal patterns, induces hydrodynamic complexities that can alter coastal circulation (de Vos et al. 2021; Franklin et al. 2025). Consequently, we hypothesized that these complexities might generate mild and localized isolation—most evident between the offshore population of Coiba and the peninsula's southern coast (Isla Cañas)—without necessarily disrupting the broader regional connectivity with populations like Chame. Nevertheless, the resolution provided by a single mitochondrial marker (COI) was insufficient to statistically confirm this specific hypothesis of isolation.

Patterns of genetic structure in *A. tuberculosa* appear to vary depending on the geographic scale. At a local scale, a study analyzing five mangrove sites within Tumaco, Colombia, reported no significant genetic differentiation (Chamorro and Rosero 2016). In contrast, at a regional scale, distinct genetic lineages have been identified between populations in northern and southern Ecuador, a pattern attributed to the Panama and Humboldt ocean currents limiting larval dispersal (Diringer et al. 2019). This context‐dependent structuring is consistent with findings in other bivalves, such as *Saccostrea echinata*, where genetic structure is shaped by a complex interaction of factors including reproductive biology, geographic isolation, and oceanographic drivers (Kim et al. 2014; Li et al. 2025; Zhang et al. 2025).

The results of the neutrality tests suggest that the populations are broadly close to genetic equilibrium, with only slight and mostly non‐significant signals of demographic expansion, with a clearer signal restricted to Chame. Specifically, Tajima's *D* showed negative values in all localities, but none were significant; in contrast, Fu's Fs was negative and significant only in Chame, reinforcing the idea of a recent population expansion at this site. These results are consistent with the haplotype network, which shows the presence of multiple unique haplotypes branching from common central haplotypes, typical of expanding populations. Additionally, the presence of unique haplotypes derived from central haplotypes shared among all populations suggests a recent divergence from a common ancestor, indicating that Panamanian populations of *A. tuberculosa* share both ancestral and more recent lineages. These patterns have been considered characteristic of species with historical demographic expansion followed by slight local differentiation (Clement et al. 2000; Leigh et al. 2015), and species with a stable demographic history and population dynamics controlled by natural gene flow (Castilla and Defeo 2005; Oyarzún et al. 2024). Therefore, the studied Panamanian populations of *A. tuberculosa* point to a dynamic evolutionary history, with high connectivity and near‐equilibrium at the local scale, but with weak and spatially heterogeneous signs of recent demographic expansion, more pronounced in Chame.

Similarly, other populations of *A. tuberculosa* in Colombia, Ecuador, and Peru show evidence of recent population expansion (Chamorro and Rosero 2016; Diringer et al. 2019). Likewise, in Chile, comparable patterns have been reported in other bivalves, such as *Mytilus chilensis* , where a haplotype network with a dominant ancestral haplotype and derived lineages was observed, indicating recent expansion from a common ancestor (Astorga et al. 2018). Specifically, Diringer et al. (2019) suggest that the demographic growth process for *A. tuberculosa* could have started at the end of the Last Glacial Maximum (LGM), which occurred about 14–15 thousand years ago in the central Pacific. Events such as the contraction of distribution range during the glacial period, followed by the appearance of favorable conditions for demographic and spatial expansion during the interglacial, have been used to explain patterns of genetic variation in other shallow‐water bivalves (Xue et al. 2014). This is because climatic fluctuations during glacial and interglacial periods, along with changes in sea level, have profoundly shaped the habitats of sessile organisms, leading to geographical isolation during sea‐level drops and favoring demographic expansion during sea‐level rises (Hu et al. 2018). In this context, a deeper genetic analysis of *A. tuberculosa* in Panama would allow us to understand key aspects of its evolutionary history, fundamental for the management and conservation of this resource.

## 5 Conclusions

The results of the present study indicate that the populations of *A. tuberculosa* in five mangroves of the Panamanian Pacific exhibit high genetic diversity, without a clear pattern of genetic differentiation among the analyzed populations, but with signs of genetic differentiation specifically between Isla Cañas and Coiba. In these localities, it is possible that the presence of ecological barriers caused by environmental gradients and geographical isolation accentuates slightly the differences in population genetic structures. Therefore, we can say that while there is high genetic connectivity among most localities, possibly mediated by larval dispersal and the influence of ocean currents, local factors that generate some degree of isolation are also at play. The coexistence of shared and exclusive haplotypes in the network, together with the clear signal of recent demographic expansion in Chame and only weak indications in the remaining populations, supports the view that these populations have maintained constant gene flow while undergoing subtle processes of local genetic differentiation. However, the inclusion of other molecular markers in the genetic analysis of this species would be very useful to strengthen this hypothesis. Despite reported reproductive alterations such as hermaphroditism, populations currently maintain high genetic diversity, likely due to the recent onset of these stressors. However, given the potential long‐term impact of self‐fertilization on heterozygosity, continuous monitoring of sex ratios alongside genetic diversity is recommended to detect early signs of variability loss.

These results have important implications for the management and conservation of the species in Panama. Our results provide a preliminary genetic baseline for *Anadara tuberculosa* in the Panamanian Pacific, indicating high connectivity among most localities, weak and spatially restricted genetic differentiation, and evidence of recent demographic expansion in Chame. From a management perspective, these patterns suggest that populations can be considered as a broadly connected management unit, in which local recruitment is supported by regional larval exchange. In this context, it is essential to avoid sharp reductions in effective population size by regulating fishing effort, enforcing minimum harvest sizes, and protecting mangrove habitats that may act as potential sources of larvae. Furthermore, we recommend integrating this genetic information with ecological and fisheries data within co‐management frameworks involving local communities, as this fishery is socially and economically important at the artisanal level. Finally, future multilocus or genomic studies (e.g., SNPs) are needed to refine the delineation of management units and to support the development of long‐term monitoring programs.

## Author Contributions

**Thalia Garcia:** data curation (equal), investigation (equal), methodology (equal), resources (equal), visualization (equal), writing – original draft (equal). **Carmen Mela‐Sánchez:** formal analysis (equal), investigation (equal), validation (equal), writing – original draft (equal). **Celestino Aguilar:** data curation (equal), software (equal), writing – review and editing (equal). **Zeuz Capitan‐Barrios:** conceptualization (equal), methodology (equal), writing – review and editing (equal). **Angel Vega:** conceptualization (equal), data curation (equal), funding acquisition (equal), investigation (equal), methodology (equal), writing – original draft (equal). **Leyda Abrego:** conceptualization (equal), formal analysis (equal), investigation (equal), methodology (equal), supervision (equal), writing – review and editing (equal). **Yolani A. Robles P.:** conceptualization (equal), formal analysis (equal), funding acquisition (equal), investigation (equal), methodology (equal), project administration (equal), validation (equal), writing – review and editing (equal).

## Funding

This work was supported by SENACYT grant FID22‐102, Fundación Isla Secas and SNI‐SENACYT awarded to L.A.

## Conflicts of Interest

The authors declare no conflicts of interest.

## Supporting information


**Supporting Information:** ece373396‐sup‐0001‐SupplementaryMaterial.xlsx.

## Data Availability

The raw data supporting the main results of the study are in the Genbank repository (accession numbers: PV450202– PV450381).
